# Modeling the Implementation Context of a Telemedicine Service: Work Domain Analysis in a Surgical Setting

**DOI:** 10.2196/26505

**Published:** 2021-06-21

**Authors:** Hedvig Aminoff, Sebastiaan Meijer, Urban Arnelo, Kristina Groth

**Affiliations:** 1 Biomedical Engineering and Health Systems KTH Royal Institute of Technology Stockholm Sweden; 2 Department of Surgical and Perioperative Sciences Surgery Umeå University Umeå Sweden; 3 Division of Surgery Department of Clinical Science, Intervention and Technology Karolinska Institutet Stockholm Sweden; 4 Innovation Center Karolinska University Hospital Stockholm Sweden

**Keywords:** telemedicine, telementoring, implementation context, surgical guidance, health technology, usability, work domain analysis, cognitive work analysis

## Abstract

**Background:**

A telemedicine service enabling remote surgical consultation had shown promising results. When the service was to be scaled up, it was unclear how contextual variations among different clinical sites could affect the clinical outcomes and implementation of the service. It is generally recognized that contextual factors and work system complexities affect the implementation and outcomes of telemedicine. However, it is methodologically challenging to account for context in complex health care settings. We conducted a work domain analysis (WDA), an engineering method for modeling and analyzing complex work environments, to investigate and represent contextual influences when a telemedicine service was to be scaled up to multiple hospitals.

**Objective:**

We wanted to systematically characterize the implementation contexts at the clinics participating in the scale-up process. Conducting a WDA would allow us to identify, in a systematic manner, the functional constraints that shape clinical work at the implementation sites and set the sites apart. The findings could then be valuable for informed implementation and assessment of the telemedicine service.

**Methods:**

We conducted observations and semistructured interviews with a variety of stakeholders. Thematic analysis was guided by concepts derived from the WDA framework. We identified objects, functions, priorities, and values that shape clinical procedures. An iterative “discovery and modeling” approach allowed us to first focus on one clinic and then readjust the scope as our understanding of the work systems deepened.

**Results:**

We characterized three sets of constraints (ie, facets) in the domain: the treatment facet, administrative facet (providing resources for procedures), and development facet (training, quality improvement, and research). The constraints included medical equipment affecting treatment options; administrative processes affecting access to staff and facilities; values and priorities affecting assessments during endoscopic retrograde cholangiopancreatography; and resources for conducting the procedure.

**Conclusions:**

The surgical work system is embedded in multiple sets of constraints that can be modeled as facets of the system. We found variations between the implementation sites that might interact negatively with the telemedicine service. However, there may be enough motivation and resources to overcome these initial disruptions given that values and priorities are shared across the sites. Contrasting the development facets at different sites highlighted the differences in resources for training and research. In some cases, this could indicate a risk that organizational demands for efficiency and effectiveness might be prioritized over the long-term outcomes provided by the telemedicine service, or a reduced willingness or ability to accept a service that is not yet fully developed or adapted. WDA proved effective in representing and analyzing these complex clinical contexts in the face of technological change. The models serve as examples of how to analyze and represent a complex sociotechnical context during telemedicine design, implementation, and assessment.

## Introduction

This paper focuses on a successfully trialed telemedicine service for remote surgical guidance [1] that was to be scaled up to four additional hospitals and clinically evaluated. However, there were many technical, social, and organizational differences between the participating clinics, and indications that the acceptance of teleguidance varied [2]. We wanted to account for the implementation context by conducting a work domain analysis (WDA) to systematically investigate what set the sites apart and identify the factors that might come to affect the implementation and clinical outcomes of the telemedicine service [3-5].

### Background

Health technology innovations that appear successful in one setting can produce different outcomes in another context. This may contribute to variability in clinical outcomes and cause failure to scale. There is growing recognition that the complexity of health care presents challenges for evaluating new health information technology (IT) [6] and that high-quality design and evaluation requires considering the context in which new technologies will be used. This paper focuses on systematically charting the implementation context for a telemedicine service for surgical consultation.

The telemedicine service was a practitioner-to-practitioner videoconferencing system designed to enable remote surgical guidance in endoscopic retrograde cholangiopancreatography (ERCP), a technically advanced procedure for biliary and pancreatic disease. The telemedicine innovation, called teleguidance, was successfully trialed through collaboration between a high-volume clinic at a university hospital and a low-volume regional clinic, and health economic modeling demonstrated positive quality impacts [7]. Teleguidance was subsequently scaled up to four additional hospitals and clinically evaluated.

However, there were many technical, social, and organizational differences between the clinics participating in the scale-up process, along with indications that acceptance toward telemedicine services varied among practitioners [2]. This raised concerns about how to successfully implement the service and understand the outcomes. It was unknown if and how contextual variations might affect clinical outcomes or whether telemedicine might interact with daily ERCP work in ways that might affect the implementation and use of teleguidance over time.

Therefore, we wanted to identify important contextual issues to be considered when evaluating the implementation and clinical outcomes by first focusing on factors that shape regular ERCP “work as done” [8] at the teleguidance implementation sites. This required a method that could accommodate the complexity of the clinical work systems [9,10]. Methodological concerns about the implementation context, complexity, and the scope of the analysis are discussed in detail in a related paper [11].

### Cognitive Systems Engineering

Cognitive systems engineering [3,12,13] is a systems design discipline for complex settings; it emphasizes that the design and evaluation of technologies must be based on knowledge about the real-world context of their use [14]. Cognitive work analysis (CWA) [3,4] is a set of methods driven by systems theory, where work systems are viewed as fields of practice in which the agents, artifacts, and external world interact to produce outcomes [15]. This set of methods has been used for design and evaluation in a range of sociotechnical systems, including health care [16].

### WDA Method

The first level of analysis in CWA is WDA. WDA is typically performed to provide representations of a complex work setting in the face of technological change (eg, during the design requirements and specification phase, or acquisition evaluation) [5]. This method has proved valuable in the design and evaluation of health technology [17], in the definition of health care team requirements [18,19], and in patient safety work [20].

WDA explicitly focuses on contextual factors by modeling the terms and conditions that shape work in functional terms. WDA provides compact representations that can support systematic investigations of how new technology impacts the overall domain purposes [21] in settings with large variability in behaviors and events as well as during system change [22].

### Objectives

Our aim was to identify intrinsic constraints that shape ERCP work from a clinician’s perspective, ranging from physical objects to processes and priorities that affect regular work. A broad WDA would provide a systematic description of the factors shaping regular ERCP work at one hospital, including the macroscopic, mesoscopic, and microscopic levels of the system [23], which are commonly analyzed and represented separately [24]. The graphical format would be useful to contrast the work systems where teleguidance is to be implemented and proactively identify how the telemedicine service might interact with work at the different sites.

The *Methods* section describes how we modeled daily ERCP work as a work system involving physical components, processes, and goals and intentions, and how this allowed us to contrast the implementation contexts at the hospitals involved and reflect upon how teleguidance might interact with the work systems.

## Methods

### Data Collection

**Figure 1:**
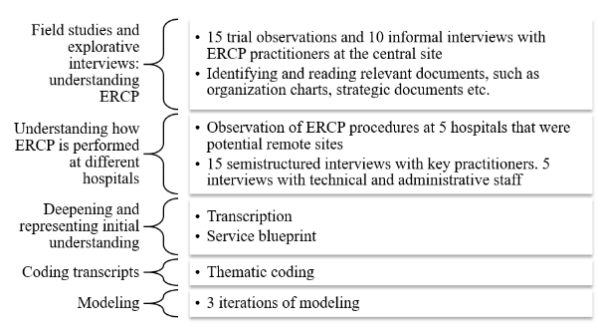
Phases in data collection, analysis, and modeling.

An ethnographical approach was used, with extensive field work conducted to collect data and generate a deep understanding of the context in a work system [25]. This included three iterations of data collection using a sequence of techniques, moving from a general “rough” level of description to a finer understanding, as Figure 1 shows.

The focus during the first round of observations and interviews was on the practical aspects of ERCP work, namely identifying everyday work practices, including tradeoffs and challenges encountered by the staff. The aim was to understand the ERCP procedure itself, roles of different stakeholders, and details about the clinical work from the perspective of the ERCP staff. We also read the clinical decision support and strategic documents and spoke to practitioners with administrative and management roles to gain an understanding of the organizational issues shaping the clinical work.

After these steps, a service blueprint [26] was developed as an intermediate, shared representation to externalize our understanding of the different phases of regular work practices and tasks during each phase (see Multimedia Appendix 1, which is in Swedish). The service blueprints were used as a resource to support discussions with practitioners and help compare work practices at the different hospitals.

The second phase of data collection included designing a protocol for semistructured interviews focusing on the details of regular ERCP practices (see Multimedia Appendix 2) and conducting a series of interviews with physicians, nurses, and technical and administrative staff at each of the four remote participant sites. Observations of the work practices, such as how surgery was prepared and conducted at each site, were documented as field notes, and the surgical facilities at the remote sites were documented as images to obtain details regarding the layout and medical equipment available in the operating theaters.

### Analysis

All interviews were recorded and transcribed. The coding work was mainly performed by the first author, with support from the second author. We conducted thematic analyses [27] to identify the constraints mentioned in the interviews; the initial codes were generated by grouping and naming interesting or repeated findings, such as the patterns of activities or the mention of challenges in the work environment or during ERCP. We used the prompts derived from the WDA framework to link our findings to a priori identified themes in line with the abstraction levels suggested by Naikar [5]( see Multimedia Appendix 3).

### Modeling

One of the common WDA representations is the abstraction hierarchy (AH). The AH matrix is a way of modeling the work domain, and it shows means-ends relationships among constraints (eg, how a physical object serves or interferes with system objectives).

The AH can be used as a tool to trace how introducing new technologies and work processes can interact with numerous aspects of work [28]. The AH was constructed using Naikar’s method [5] as the main resource, together with feedback from three domain experts, two ERCP surgeons and one project manager.

The modeling focused on the ERCP team subsystem, and these cells were populated at the highest level of detail in the AH matrices.

Suggestions regarding system decomposition and populating the cells of the AH were developed through multiple iterations, and the details are available elsewhere [11].

We worked through several versions of the work system decomposition, identifying systems and subsystems within the hospital organization. We found that the open nature of the hospital systems and constant reorganization made it difficult to define a detailed hierarchical decomposition that would contribute to the analysis. Moreover, after three modeling iterations, we found a satisfactory way to represent the domain as three functional facets: treatment, development, and administration.

These facets are sets of constraints distinguished by the nature of the tasks, competencies, and roles. Individuals can have multiple roles and be involved with several facets, as is the case with senior physicians and nurses who may perform clinical, managerial, research, or teaching/mentoring functions.

### Exploring Interactions Among Constraints

**Figure 2:**
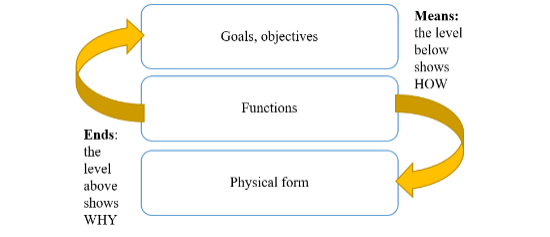
How the means-ends structure of an abstraction hierarchy can support the investigation of interactions in a work system.

Considering the individual nodes in the AH and tracing the means-ends links to the levels above and below (Figure 2), the models were used as a tool to verify our understanding of how ERCP is currently performed and further explore possible interactions and system changes when teleguidance is introduced.

The purpose of these exercises was to elicit new insights about the implementation of teleguidance and determine whether the models provided a representation that different stakeholders could relate to.

## Results

### Multiple Models

Owing to the open nature of the work systems, the scope of our analysis was very wide and deep; we identified many causal (physical) and intentional constraints (goals, priorities, etc). Some constraints were conflicting, such as policies that might cause tradeoffs between clinical performance and economic efficiency.

**Figure 3:**
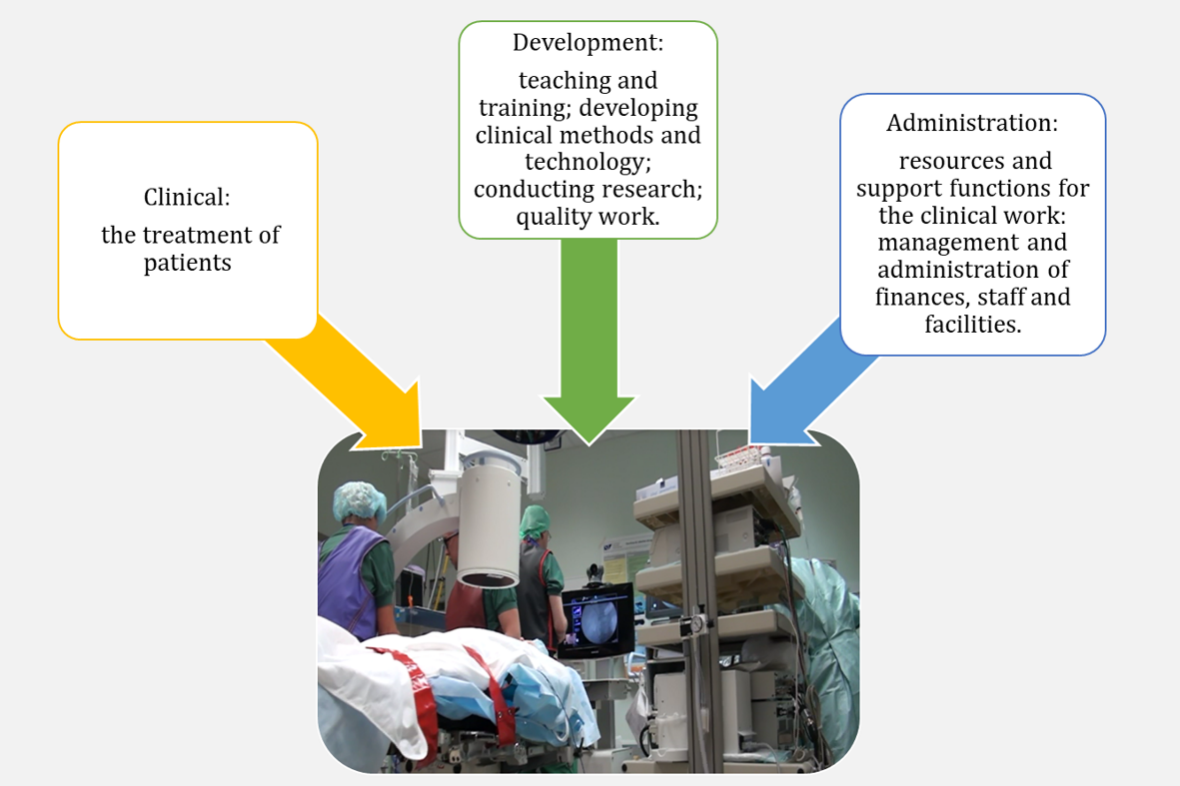
Three sets of constraints shaping daily endoscopic retrograde cholangiopancreatography work.

The complexity of the context was difficult to incorporate within a single AH, and we resolved this by modeling three sets of constraints affecting ERCP procedures, namely the clinical, administrative, and development facets of the domain, as Figure 3 shows.

In the following sections, we present the AHs, which were aggregated to increase legibility, and representative examples of constraints; we describe how these can vary between the implementation sites and how the constraints may interact with teleguidance. The clinical facet is described in greater detail than the development and administration facets.

### The Clinical Facet

**Figure 4:**
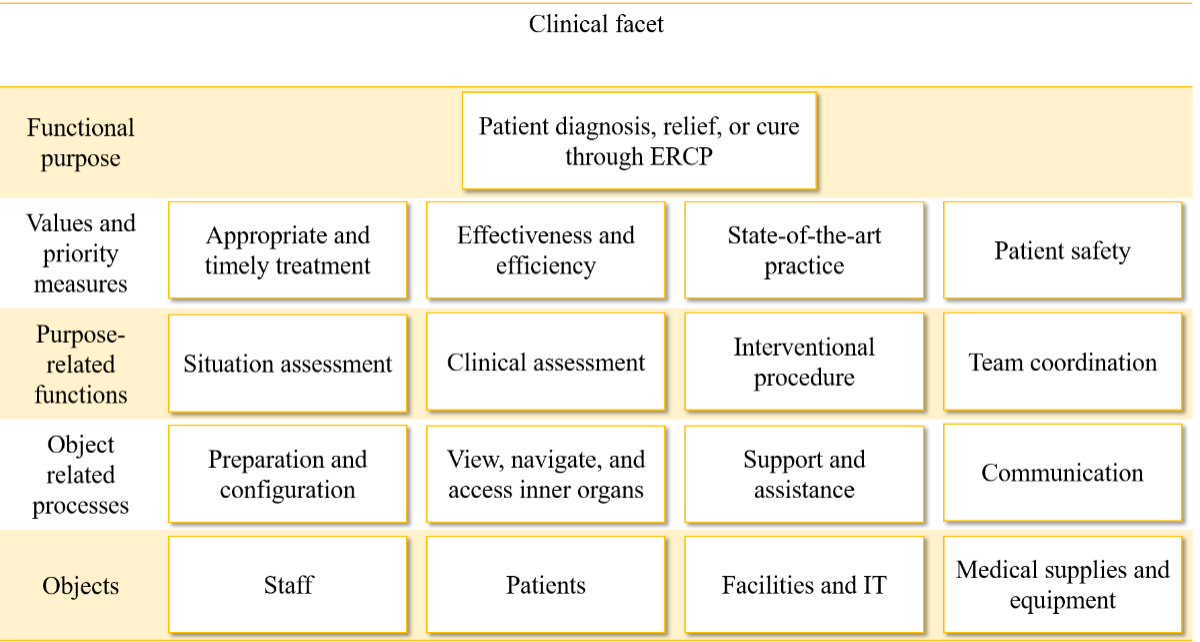
Abstraction hierarchy, clinical facet.

The clinical facet (Figure 4) represents the constraints that shape the ERCP team’s work in terms of the functional purpose, namely “patient diagnosis, relief, or cure through ERCP.” The physical entities are the ERCP team members, patients, medical facilities, and medical equipment.

The set of possible clinical actions during an ERCP session is largely determined by causal constraints such as the patient’s condition, staff competencies, and capabilities and limitations of the available technology and devices. Teleguidance introduced new physical equipment and staff to the ERCP team, providing new affordances and constraints that propagate throughout the clinical facet.

Constraints were represented in considerably higher detail during modeling. Multimedia Appendix 4 shows a cropped image of a detailed model of the clinical facet.

### Values and Priority Measures

The values and priority measures show the criteria that must be respected for the clinical facet to achieve its functional purposes and those guiding decision-making and tradeoffs during procedures (eg, between patient safety and quality on the one hand, and efficiency on the other hand).

We visualized four values and priority measures: appropriate and timely treatment; effectiveness and efficiency; state-of-the-art practice; and patient safety (see Multimedia Appendix 5).

Multimedia Appendix 6 shows an example of how “appropriateness,” a value/priority constraint, differs among hospitals, and how this might interact with teleguidance. Textbox 1 shows an example transcript regarding findings linked to the abstracted constraint “appropriateness.”

**Sample transcript showing value and priority measures: appropriate treatment.**

**Appropriate treatment**

“Yes, it is more dangerous with PTC* than ERCP, even though ERCP can be dangerous and risky. But I have noticed it all over the country so there are still ideas about PTC and if you are not as skilled at ERCP then it often happens that you do them.”

“We have shown it, the more skilled we become at ERCP the fewer the PTCs will be. Provided the indications are the same. But in the past people were allowed to die in icterus. And that's not so very long ago. And it may not be so... if you have a huge spread of cancer, then maybe you should ask yourself how far to drive this? The oncologists are happy to push for it only if there is a small snippet left to live, to try an option. But we still have to consider, I want to do that, what do we want, what is the goal of this activity? After all, the person will die within a month or so anyhow… maybe it's not that bad to die of icterus instead of drying out emaciation, pain everywhere.”

*Percutaneous transhepatic cholangiography and drainage

### Purpose-Related Functions

The purpose-related functions represent the general functions that the ERCP work system must fulfill to achieve its functional purpose.

We defined four main functions: situation assessment, clinical assessment, interventional procedure, and team coordination (see Multimedia Appendix 7).

Multimedia Appendix 8 illustrates how this constraint shapes work, how it can vary between sites, and how it might interact with teleguidance.

### Object-Related Processes

The object-related processes level represents the functional capabilities of physical objects, namely the use of physical objects, and their properties and affordances.

We defined four main object-related processes: preparation and configuration; view, navigate, and access inner organs; support and assistance; and communication (see Multimedia Appendix 9).

Multimedia Appendix 10 provides an example of the constraint “preparation and configuration.”

### Physical Objects

The physical objects level shows objects that afford functional capabilities to the system. Causal constraints such as patient conditions, staff availability and competencies, and the capabilities and limitations of the technology and devices in an ERCP clinic constrain the set of possible clinical actions during an ERCP session.

We grouped the large number of physical objects required during ERCP procedures into four main categories: staff; patients; facilities and IT; and medical supplies and equipment (Multimedia Appendix 11).

Multimedia Appendix 12 provides an example of the constraint “facilities and IT.”

### The Administrative Facet

**Figure 5:**
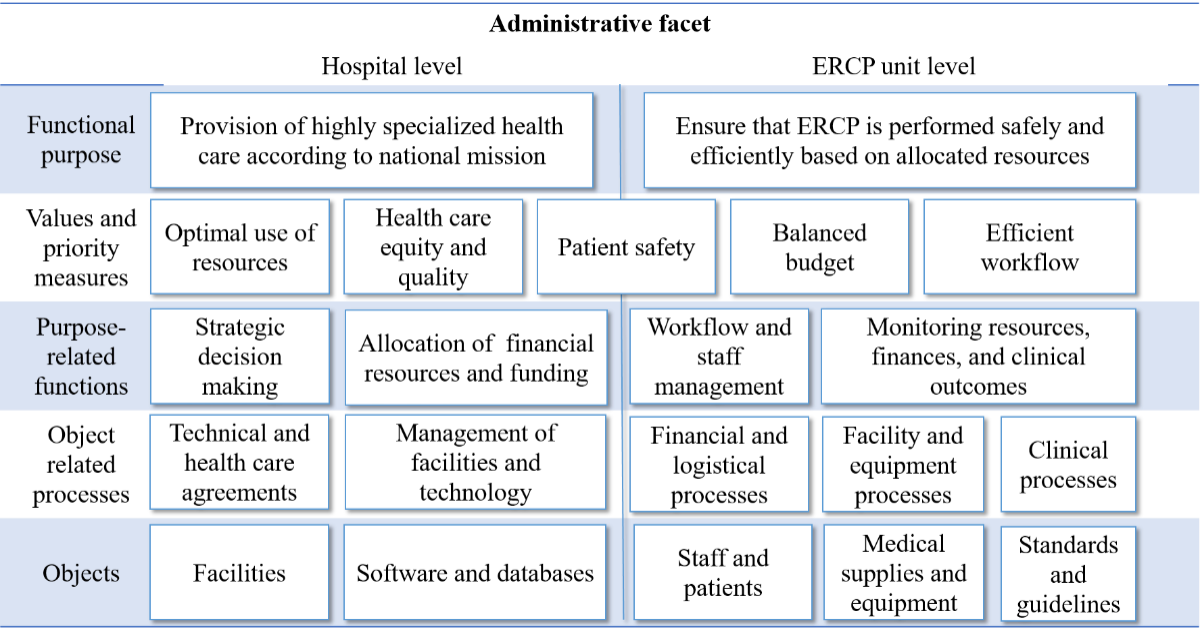
Abstraction hierarchy, administrative facet.

The administrative facet (Figure 5) was conceptualized as part of the domain that provides the resources for “primary” clinical work.

This facet is largely shaped by intentional constraints such as institutional objectives, organizational and management policies, legislations, and regulations. The administrative facet also places demands on work performance, such as efficiency. Although efficiency demands are considered even during surgery, these demands are largely controlled from outside the clinical facet, which is emphasized in our model.

Individuals can play multiple roles and be involved with several facets, as is the case with senior physicians and nurses who may be directly involved in an ERCP procedure (clinical facet) while also executing managerial, research, and teaching/mentoring functions.

We arrived at two levels of decomposition when we modeled the university hospital, one at the hospital level and the other involving the subsystem “ERCP unit.” Although the administrative facet largely set intentional constraints on ERCP work, there were also causal constraints that might affect teleguidance.

All hospitals were under considerable pressure for increasing their efficiency and undergoing constant reorganization. At the hospital level, this was represented as the values and priority measure “optimal use of resources,” and at the unit level, it was represented as “balanced budget” and “efficient workflow.”

Multimedia Appendix 13 shows an example of how the purpose-related function “strategic decision-making” in the administrative facet might affect teleguidance. More examples are presented in Multimedia Appendix 14.

### The Development Facet

**Figure 6:**
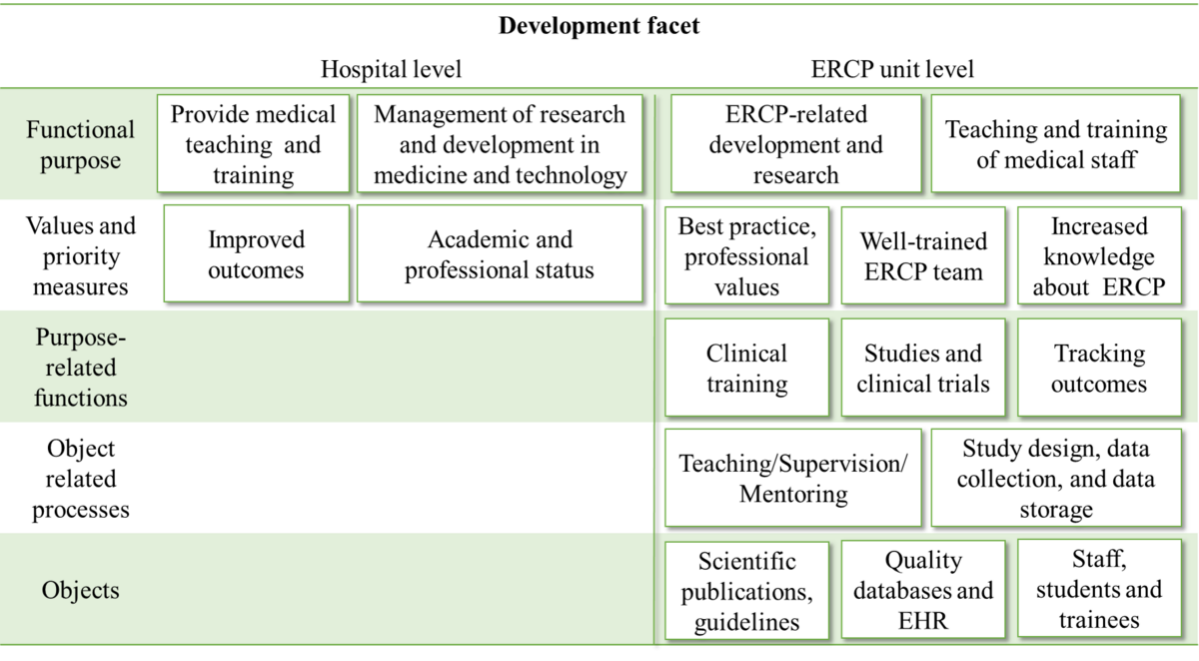
Abstraction hierarchy, development facet.

The development facet (Figure 6) is distinguished from the administrative facet owing to its focus on training, research, and quality management, which are the characteristics of advanced clinical practices. In many cases, funding and accountability for these activities are external to the ERCP work system; clinical education and training are often linked to an educational facility; research funding is external; and clinical quality and patient safety criteria are set according to standards and regulations. However, many development activities take place during procedures, such as research activities (eg, documenting unusual physiological features) or teaching and training activities (eg, taking extra time for instruction or allowing a less experienced practitioner operate equipment under supervision).

The development facet is accorded higher priority at the university hospital, where part of the functional purpose includes a mission to provide development, education, and training at the national level.

Many aspects of the development facet appeared to motivate the staff we interviewed, and academic and professional statuses were important aspects for being regarded as trustworthy team members. At the regional hospitals, there were expectations that teleguidance would strengthen the development facet by not only increasing opportunities for training but also for participating in research activities.

Multimedia Appendix 15 provides an example of how a constraint in the development facet might affect teleguidance.

## Discussion

### Principal Results

The ERCP work system is an open system as there are factors beyond the control of the clinical team, which influence how ERCP procedures are conducted. The high number of factors that could potentially affect each procedure led us to characterize the ERCP work context as having three facets, representing clinical work as the primary field of interest, and administration and development as complementary fields of the domain, which provide resources for clinical work.

Each facet served as a “dimension” along which we could reason about the differences between the implementation sites and how the different sets of constraints affecting ERCP could impact the implementation of teleguidance.

Although the clinical and administrative facets in some aspects reflected organizational partitions, the development facet was clearly not reflected within the organizational structures. However, development is an important aspect of the work domain, which motivates staff and shapes daily clinical work in the highly specialized and constantly evolving field of ERCP, where quality work, training, research, and design of medical equipment are pervasive.

### Specific Findings

The implementation sites could be described with the same AHs and compared by contrasting how specific constraints were instantiated.

The functional purposes of the clinical facet show the ERCP team’s primary objectives, namely “patient diagnosis, relief, or cure through ERCP.” Teleguidance will not change the functional purpose, but it will affect the constraints through which this purpose is achieved.

During procedures, we expect teleguidance to mainly support the purpose-related functions of clinical assessment and interventional procedures by advising how to interpret imagery or providing specific suggestions for handling a certain instrument.

Teleguidance may create challenges in the functions of “situation assessment” and “team coordination.” Situation assessment will change in some ways during teleguidance sessions because team members will be in different locations, and there are risks that the remote surgeon and on-site team might perceive the situation differently (eg, the guiding surgeon may miss information that is apparent to the on-site team).

Team coordination may be affected in ways we do not yet understand as the guiding surgeon becomes part of a geographically and organizationally distributed clinical team that requires cognitive, practical, and administrative coordination.

Teleguidance may also cause differences in the values and priorities among clinical practitioners to surface during teleguidance sessions, as shown by the example of appropriateness.

The broad definition of the ERCP work system allowed us to identify constraints that shape events during procedures, along with the object-related processes included in in the work prior to the clinical procedure, such as preparation and configuration. An example is how handling the telemedicine equipment can affect the workload of the assisting staff; at two of the hospitals, the staff would need to spend additional time for preparation and configuration as they must fetch and set up the teleguidance cart, as well as establish a functioning video link. In an already strained work setting, this inconvenience may very well lead to negative experiences with teleguidance over time.

Regarding the “secondary” facets of the domain, the AHs provide some indications of how technical and administrative issues may play out more significantly over time, such as technical responsibility for service and reimbursement issues. We expect that the initial mismatches between the administrative facets can be overcome if the priorities and responsibilities for development work are clear.

If development activities are not a priority, then there is a risk that users will not have the time and resources necessary to handle the awkwardness of work process adaptations.

### Comparison With Prior Work

There are prior examples of WDA that distinguish primary operations and resource management through multiple models, showing the different stakeholder perspectives or facets of a problem [5]. There are also examples of behavioral studies involving health care work systems, which differentiate clinical work and the infrastructure and resources for this work, conceptualizing health care work in terms of primary (clinical) and secondary (billing, audit, and management) work activities [29].

In our case, a third facet, development, is relevant, and this highlights that teleguidance is an effort to facilitate training and quality assurance in routine clinical ERCP practices and the control and constraints for these aspects appear different from the clinical and administration facets.

### Limitations

There are many ways to construct AHs, and the answer to the question of whether it is well done in this study lies in issues such as the boundary definition and conceptualization of abstraction levels, goals, and objects. These aspects were continually addressed during the numerous iterations for creating the AHs.

Owing to the broad system definition and the open nature of the work system, it was not easy to achieve a hierarchical decomposition, and we initially struggled to represent the wide array of constraints. The facets presented a solution to this dilemma.

WDA is developed for complex settings that are resistant to deterministic analysis because of their nature. Therefore, AHs cannot be objectively correct or complete; the highlight of this study is that the models provide a structured and accountable way to reason flexibly and imaginatively on how constraints from multiple system levels interact.

### Conclusions

According to numerous reviews and policy documents, system dynamics and complexity should be considered during the design and evaluation of technological change in health care [30]. This includes the contextual factors constituting “the normal conditions of practice,” thus contributing to the implementation outcomes [31]. Our WDA serves as an example of how a complex clinical implementation context can be analyzed and represented in a in a granular yet structured manner while also showing the interactions among the system elements.

We identified clinical, development, and administrative facets of the work domain. These facets represent the general aspects of clinical work systems as sets of contextual factors that should be factored in during the design and implementation of any telemedicine service.

### Future Work

The AHs can serve as artifacts to support the shared understanding required for multidisciplinary collaboration, which is a prerequisite for successful human-system integration [32] (eg, by increasing project managers’ understanding of project complexities). This may be extremely valuable in participatory development processes such as contextual inquiries and value specifications, which are important for developing a holistic implementation approach [33].

The models may also be valuable for providing the necessary insights regarding proactive project risks and patient safety management during implementation [34] as well as for guiding clinical and project evaluations [11].

## Appendix

Multimedia Appendix 1 Service blueprint showing the processes involved prior to, during, and after endoscopic retrograde cholangiopancreatography (in Swedish).

Multimedia Appendix 2 Protocol for semistructured interviews. ERCP: endoscopic retrograde cholangiopancreatography.

Multimedia Appendix 3 Coding categories and criteria for qualitative data analysis. ERCP: endoscopic retrograde cholangiopancreatography.

Multimedia Appendix 4 Partial view of a detailed model of the clinical facet. This image shows the high level of detail in which we modeled the domain, prior to aggregating the abstraction hierarchies.

Multimedia Appendix 5 Values and priority measures.

Multimedia Appendix 6 Example of the value/priority constraint “appropriateness” showing how it diverged among hospitals and how this might interact with teleguidance. ERCP: endoscopic retrograde cholangiopancreatography.

Multimedia Appendix 7 Purpose-related functions.

Multimedia Appendix 8 Example of purpose-related function: team coordination. ERCP: endoscopic retrograde cholangiopancreatography.

Multimedia Appendix 9 Object-related processes. ERCP: endoscopic retrograde cholangiopancreatography.

Multimedia Appendix 10 Example of object-related processes: preparation and configuration. ERCP: endoscopic retrograde cholangiopancreatography.

Multimedia Appendix 11 Physical objects (aggregated). ERCP: endoscopic retrograde cholangiopancreatography; IT: information technology.

Multimedia Appendix 12 Example of physical objects: facilities and information technology.

Multimedia Appendix 13 Example of how the purpose-related function “strategic decision-making” might interact with teleguidance. ERCP: endoscopic retrograde cholangiopancreatography.

Multimedia Appendix 14 Examples of constraints in the administrative facet that might affect teleguidance. ERCP: endoscopic retrograde cholangiopancreatography; IT: information technology.

Multimedia Appendix 15 Example of how a constraint in the development facet might affect teleguidance. ERCP: endoscopic retrograde cholangiopancreatography.

## Abbreviations

AH: abstraction hierarchy
CWA: cognitive work analysis
ERCP: endoscopic retrograde cholangiopancreatography
IT: information technology
WDA: work domain analysis
