# Supplementary figures and images for: Modeling the Implementation Context of a Telemedicine Service: Work Domain Analysis in a Surgical Setting
Source: JMIR Form Res. 2021 Jun 21;5(6):e26505. doi: 10.2196/26505 (PMC8277332; doi:10.2196/26505)

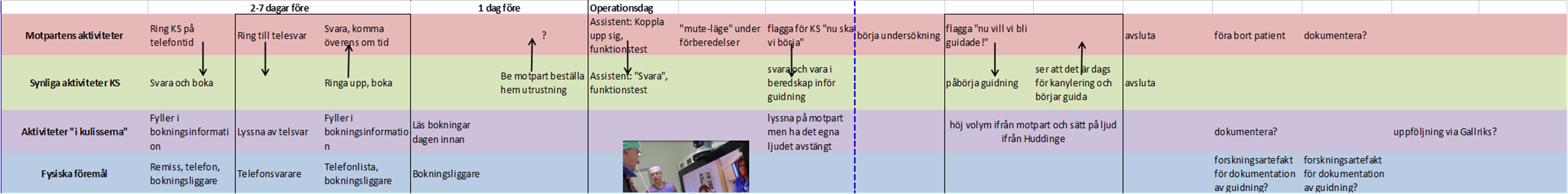

Supplement: Multimedia Appendix 1 [file formative_v5i6e26505_app1.png]

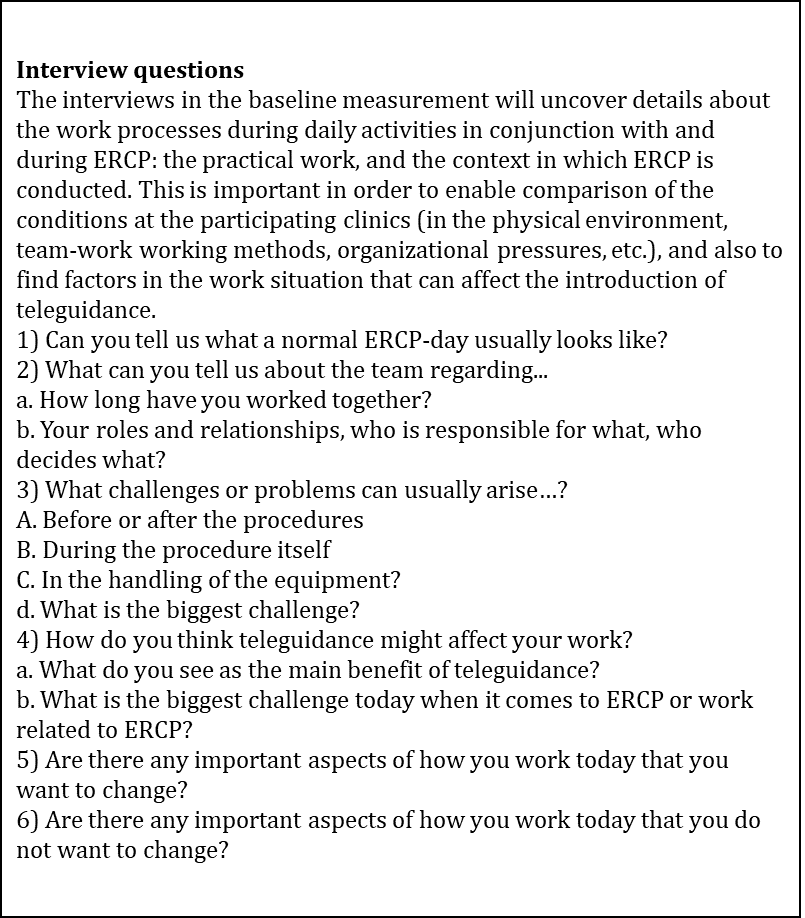

Supplement: Multimedia Appendix 2 [file formative_v5i6e26505_app2.png]

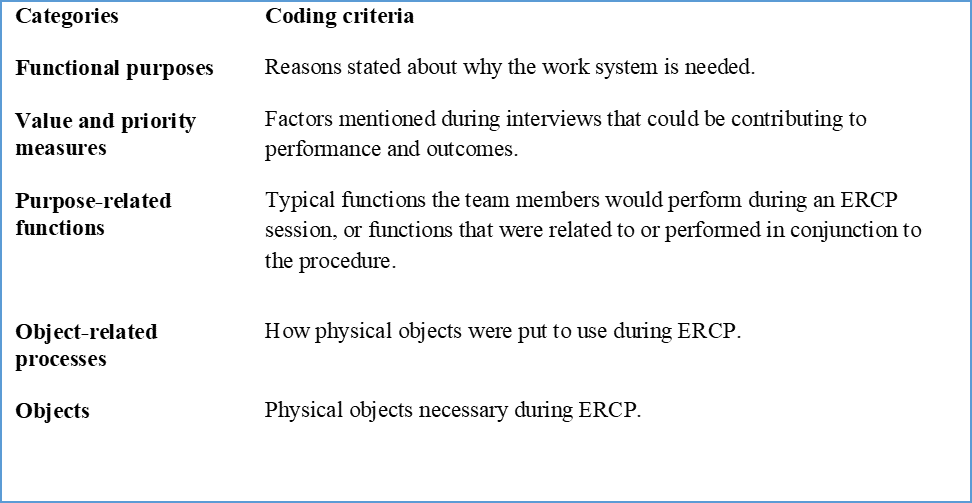

Supplement: Multimedia Appendix 3 [file formative_v5i6e26505_app3.png]

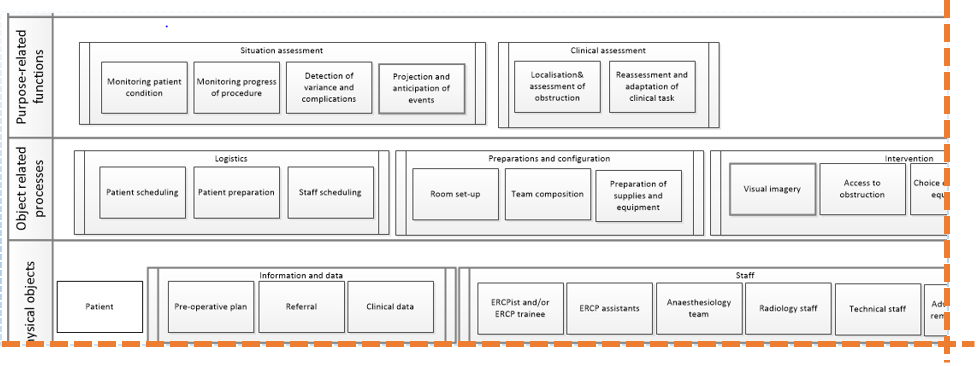

Supplement: Multimedia Appendix 4 [file formative_v5i6e26505_app4.png]

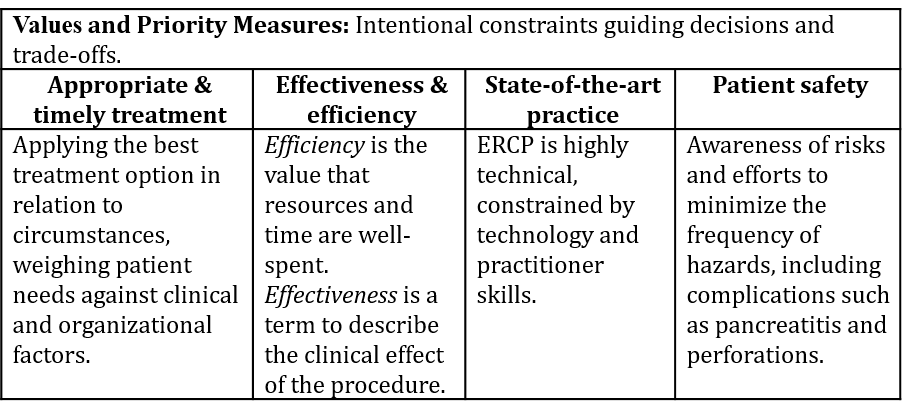

Supplement: Multimedia Appendix 5 [file formative_v5i6e26505_app5.png]

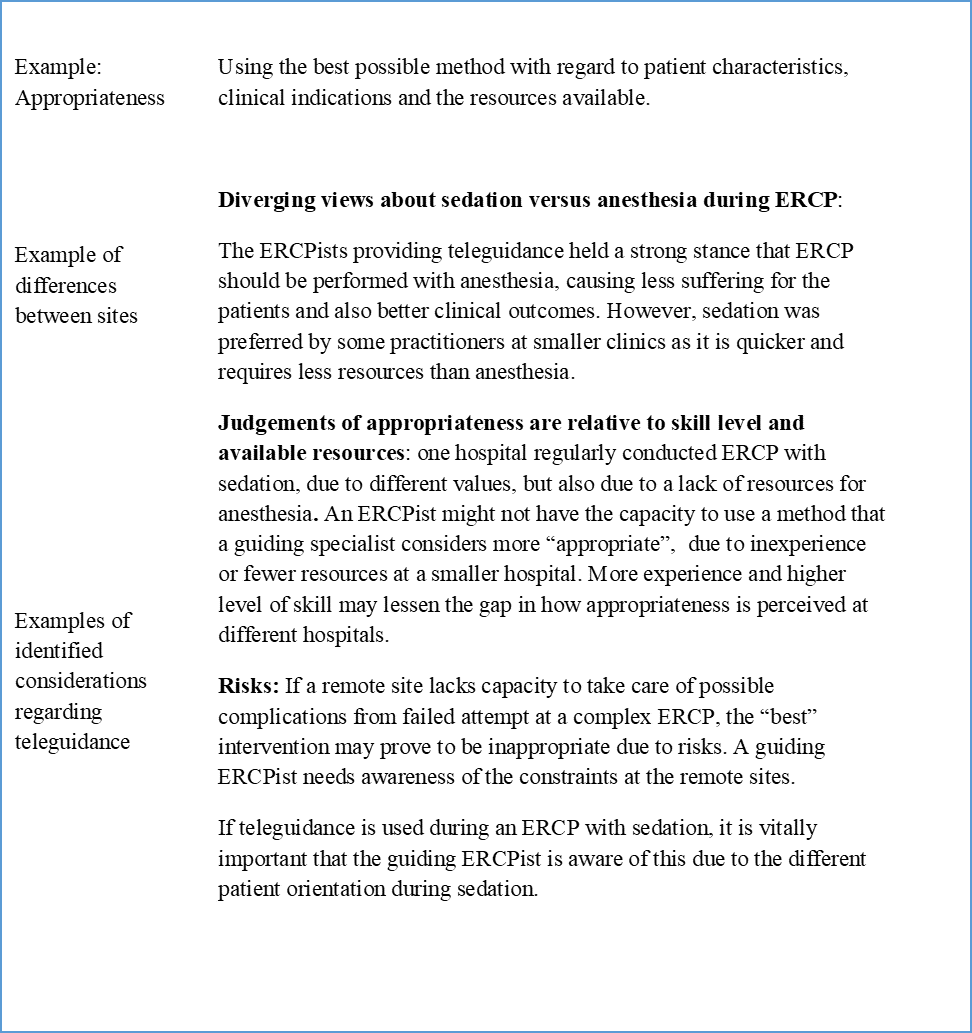

Supplement: Multimedia Appendix 6 [file formative_v5i6e26505_app6.png]

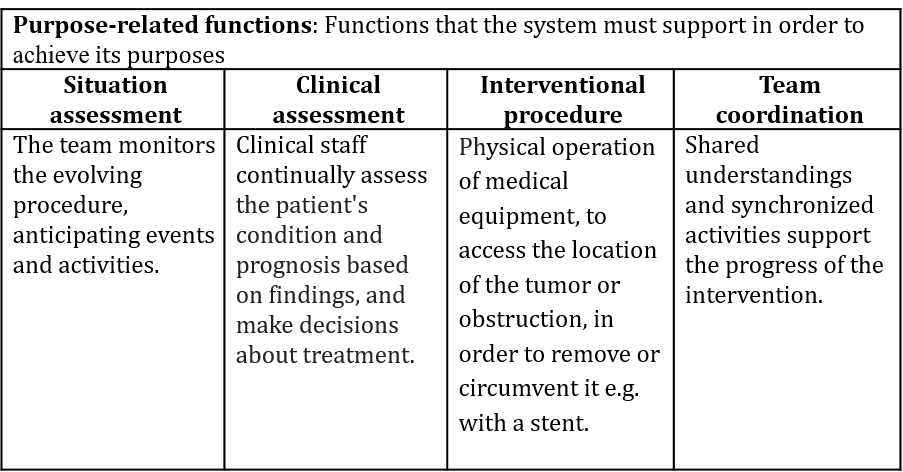

Supplement: Multimedia Appendix 7 [file formative_v5i6e26505_app7.png]

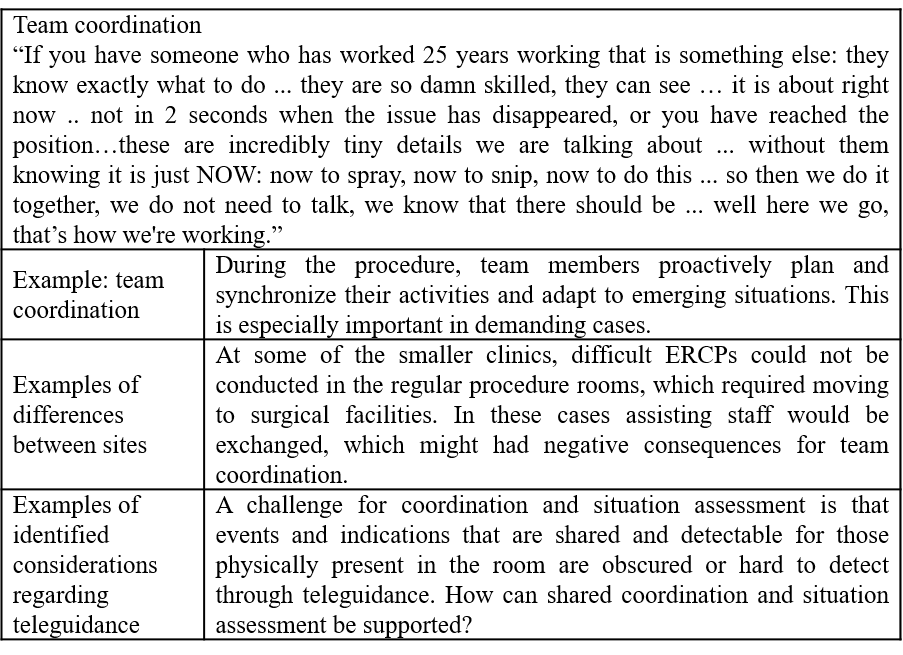

Supplement: Multimedia Appendix 8 [file formative_v5i6e26505_app8.png]

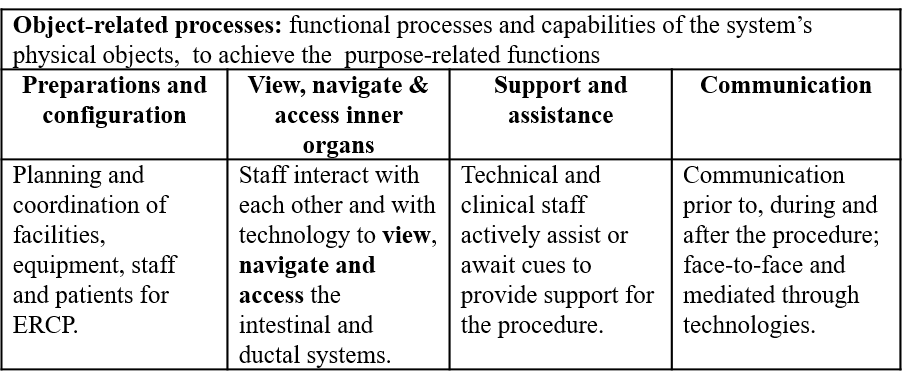

Supplement: Multimedia Appendix 9 [file formative_v5i6e26505_app9.png]

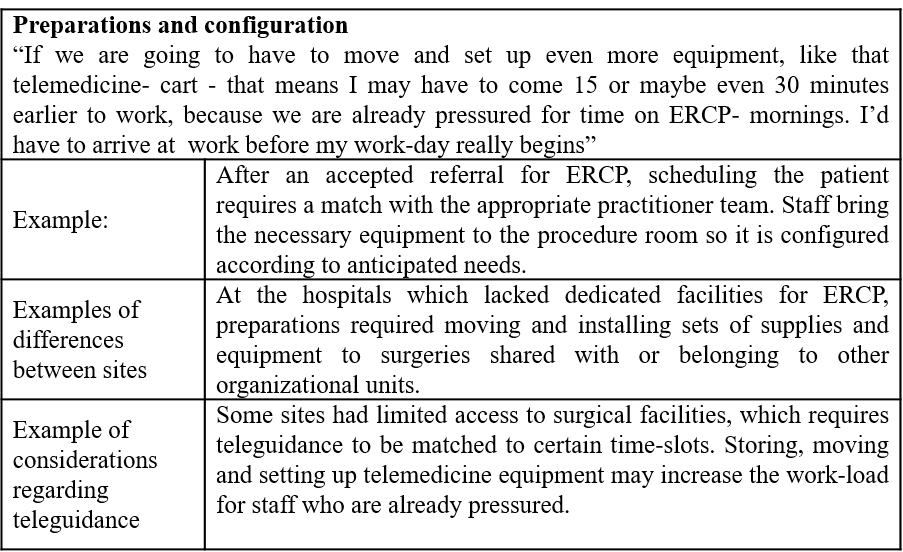

Supplement: Multimedia Appendix 10 [file formative_v5i6e26505_app10.png]

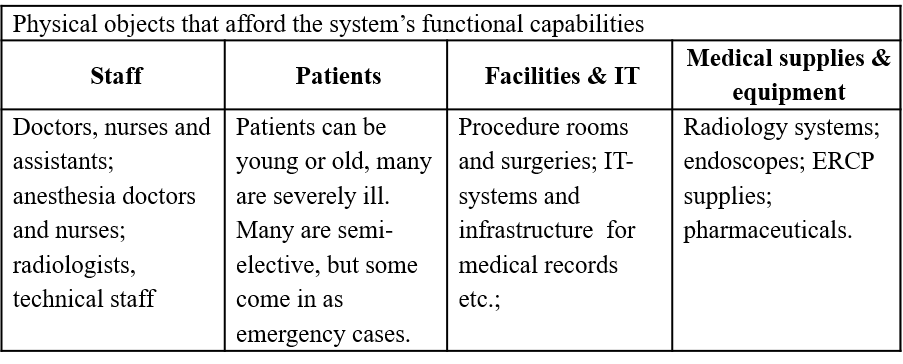

Supplement: Multimedia Appendix 11 [file formative_v5i6e26505_app11.png]

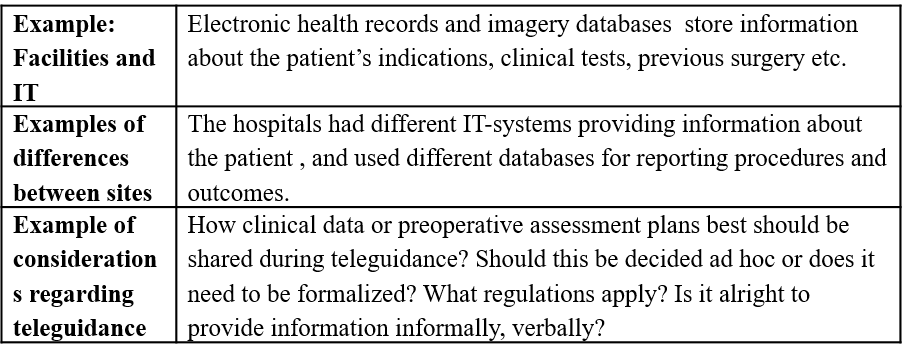

Supplement: Multimedia Appendix 12 [file formative_v5i6e26505_app12.png]

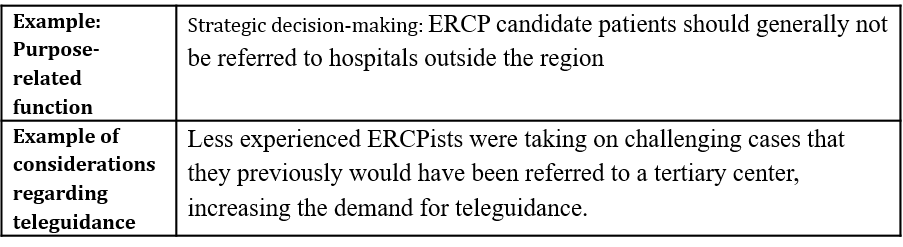

Supplement: Multimedia Appendix 13 [file formative_v5i6e26505_app13.png]

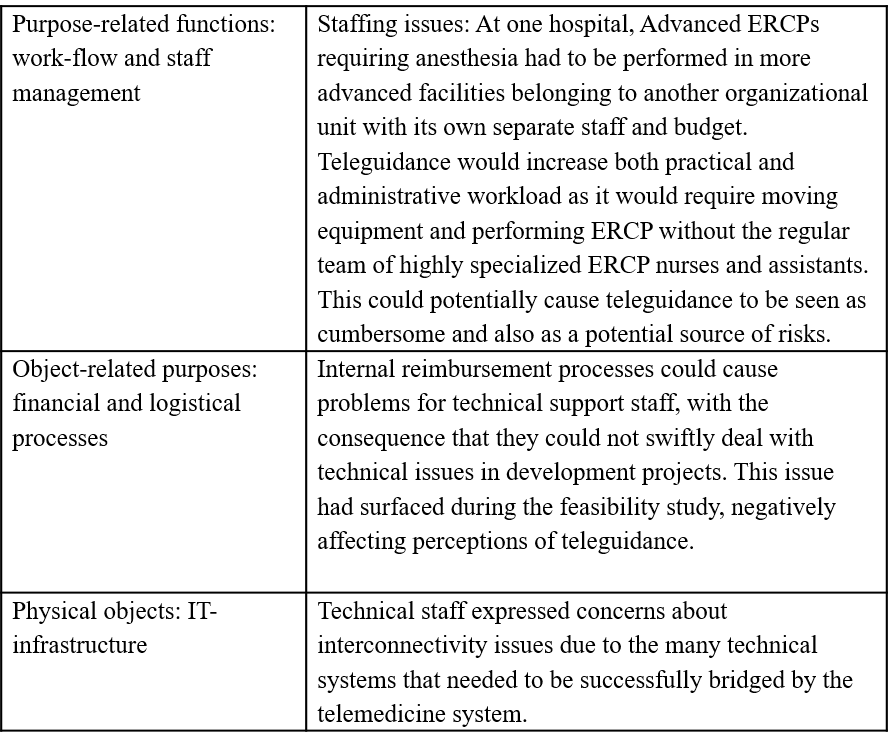

Supplement: Multimedia Appendix 14 [file formative_v5i6e26505_app14.png]

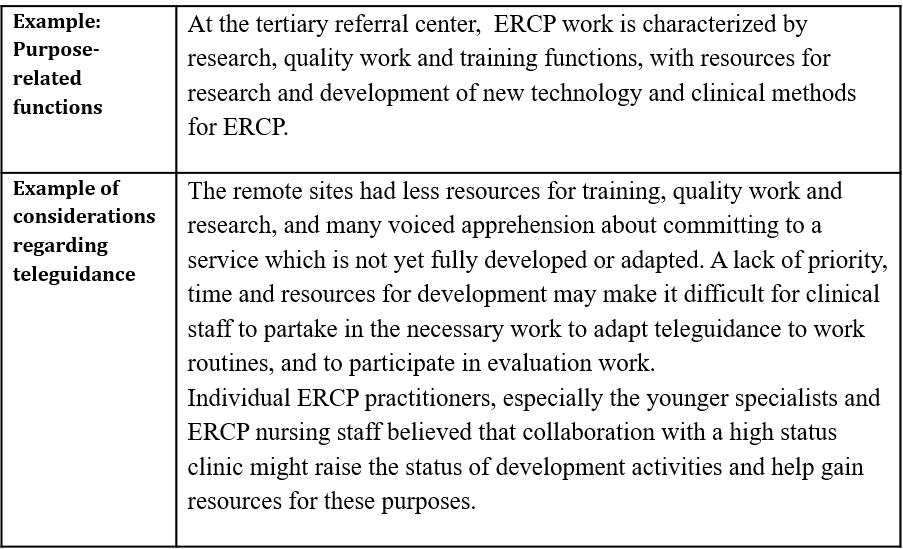

Supplement: Multimedia Appendix 15 [file formative_v5i6e26505_app15.png]
